# A Mesio-Occlusal Cavity in an Upper First Permanent Molar

**Published:** 1901-02-15

**Authors:** C. N. Johnson

**Affiliations:** Chicago, Ills.


### A MESIO-OCCLUSAL CAVITY IN AN UPPER FIRST PERMANENT MOLAR.

BY C. N. JOHNSON, L.D.S., D.D.S., CHICAGO, ILLS.

Read at the meeting of the Ohio State Dental Society, Columbus, Ohio, December 5, 1900.

The reason for selecting such a title for this paper is traceable to a request of your President that the author give a clinic upon this kind of a cavity and then outline to the Society the methods employed in the operation, together with a detailed statement of the reasons for each step in the procedure. It will be understood at the outset that the methods herein advocated, and the suggestions made in connection with the subject, apply as well to other proximo-occlusal cavities in molars and bicuspids as to the one under consideration—in so far, at least, as they are subjected to the same conditions.

When a cavity like this presents itself, there are several problems for the operator to consider. He must have sufficient separation between the molar and bicuspid to permit of so contouring the filling that the teeth are held sufficiently apart to maintain the interproximal space in its normal width and form, to the end that the gum tissue may occupy the space in a healthy condition.

If the teeth are allowed to drop too close together, so that the space is perceptibly narrowed, the gum tissue is strangled out of its normal relation to the teeth and the space left unhealthy. A case like this will present itself with the gum festoon on either side of the space standing further crownwise than normal, and in a swollen, hypertrophied condition, so as to bleed on the slightest touch. The normal form of the gum in the interproximal space is arched, with the crest of the arch reaching near the contact points on the proximal surfaces of the teeth, and sloping away from this buccally and lingually to blend with the buccal and lingual gum tissue. In case of an obliterated interproximal space, the arch of the gum is inverted so that the buccal and lingual festoons of gum stand farther crownwise than the gum immediately between the teeth—thus forming a pocket for the lodgment and retention of food between the proximal surfaces of the teeth. In the normally arched form of the gum the tendency is invariably for food which may have been forced between the teeth to be carried away out of the space along the sloping surfaces of the gum ; but when this arched form is destroyed and a pocket created, each impact of mastication merely wedges the particles of food more firmly between the teeth. This is particularly true of fibrous food, and the fibers thus held in contact with the surfaces of the teeth readily decompose and bring about decay, either in surfaces previously sound or as a recurrence of decay around fillings.

Not only this, but the constant irritation induces disease of the gum tissue and peridental membrane, sometimes even leading to the formation of a pus pocket between the teeth, with all the characteristics of pyorrhea. No operation upon these approximal surfaces should be considered perfect which does not involve the most careful attention to the form of the interproximal space and the condition of the gum tissue in it.

The next consideration in the management of these cases relates to the outline of the cavity margins. In studying the history of caries on these proximal surfaces, it will be found that there is a certain area which is exceedingly liable to decay. This area is located in the vicinity of the contact point, and it is in this region that caries usually makes its initial attack. The farther we get away from this point buccally and lingually, the less the liability to decay, and in establishing the cavity margins this fact should be borne in mind. If a permanent operation is designed, the cavity should be so extended bucco-lingually as to carry the line between filling and enamel to a point of safety. This point of safety is reached only when the margins of the filling are so situated that they are constantly kept clean by the wiping of food over them in the process of mastication, or by the friction of the cheeks or tongue againt them. The exact degree of extension necessary to accomplish this may readily be determined by the observant operator in each individual case presented, by carefully noting the conformation of the tooth to be filled and the one adjacent to it. A cavity in a molar facing the surface of a bicuspid will not require the same degree of extension that would be necessary if the cavity faced the broad proximal surface of a molar, on account of the more rounded contour of the bicuspid and the lesser width of the tooth bucco-lingually. Each case must be studied in the light of its special requirements and the marginal outline established in accordance with the existing indications.

The extension of the cavity rootwise at the gingival margin is also a question calling for careful consideration. It may be laid down as an axiom that wherever the gingival margin of a filling on a proximal surface extends under the free margin of the gum, so that the gum tissue in occupying the interproximal space covers a portion of the filling, the margin so covered is absolutely safe from a recurrence of decay, provided the filling is properly inserted and the gum tissue healthy. This would seem to argue strongly for such an extension of the cavity as to ensure this kind of protection to the filling, and unless there is some special reason against it the cavity should be so formed. This may appear like very radical teaching, but a careful study of the conditions will reveal the fact that it is not so radical as it seems. In those cases where the gum extends normally into the interproximal space, it will require but a a very slight extension of the gingival margin to carry it well under the gum, and in fact in most cases the decay has already carried it there. In other instances the cavity is found to dip down rootwise into the dentin at this point, leaving the enamel standing. This edge of enamel should never be allowed to remain in any event, and its removal will almost invariably carry the gingival margin safely under the gum. But in those cases where there has been an extensive and permanent recession of the gum, so that there is an appreciable distance between the decayed cavity and the gum margin, it would be folly to attempt to cut through sound tissue to the extent of two or three millimeters for the purpose of carrying the filling under the remaining gum. It is a question of judgment with this, as with other matters calling for discrimination in different cases, but the principle of protection to the gingival margin of the filling should never be lost sight of.

One of the chief considerations connected with this kind of cavity relates to the form that shall be given it for the most secure retention of the filling. When the aggregate impact brought to bear upon such a filling in mastication is carefully considered, it will be apparent that in order to anchor it securely against displacement the cavity must be formed upon scientific principles and according to mechanical rules. Few dentists really stop to consider the exact nature of the service their fillings are likely to be called upon to perform. It has been somewhat conservatively estimated that during the mastication of an ordinary dinner the jaws are brought into occlusion at least one thousand times. The force of these occlusions may vary in different mouths, and also according to the character of the food, but to penetrate an ordinary piece of beefsteak with the teeth will require—according to Dr. Black—at least sixty pounds pressure. Supposing only one-fourth of the entire number of occlusions chance to fall upon the filled tooth, it will readily be seen that the aggregate number of impacts against the filling in the course of a year will be something enormous. There are, of course, many fillings in which the relation of the cusps of the opposing tooth is such that very little direct impact is brought to bear upon them, and almost any kind of anchorage will be sufficient to hold the fillings in place. It is just such cases as these that mislead many operators into a false security as to the character of anchorage necessary to retain fillings against displacement.

In every instance where an opposing cusp impinges directly against the filling, as is usually the case with a normal occlusion, the cavity must be so formed as to retain the filling against severe usage, extending over a number of years. To accomplish this, due consideration must be had for mechanical principles. The portions of the cavity against which the filling is driven under the impact of mastication should be made flat instead of concave, and should be formed as nearly as possible at direct right-angles to the force of occlusion. To this end the gingival wall should be horizontal from buccal to lingual, and from the gingival enamel margin to the axial wall. If any deviation is made from this, it should be to dip it slightly root-wise, as it approaches the axial wall, giving it a dovetailed effect to lock the filling more securely into place. The anchorage step on the occlusal surface should also be formed with a flat base, which should join the surrounding walls of the step at right-angles. This flattening of those walls of the cavity which are to receive the principal impact adds very materially to the stability of the filling under stress, and prevents tipping or rocking of the filling while it is being built.

The formation of angles to join the walls of the cavity is also an element of security. The gingival wall should join the axial wall at a direct right-angle, and there should be a point angle at the gingivo-axio-buccal and gingivo-axio-lingual corner of the cavity. This point angle should be extended into a line angle between the axial and buccal and the axial and lingual walls, as far toward the occlusal as can be done without undermining the cusps. No angles should be made along the marginal outline of the cavity. The gingival enamel margin should be flat bucco-lingually, and the buccal and lingual margins as nearly perpendicular as possible, but the gingival enamel margin should be made to join the buccal and lingual margins, not at an angle, but on a short curve. This is true of all marginal outlines.

The formation of angles in the interior of cavities has been criticised for two reasons. First, because of the supposed difficulty of adapting filling materials into angles, and, second, because of the fear of weakening the tooth. The claim has been made that a fracture will occur more readily at an angle than at any other point, and that the formation of angles between walls invite the fracture of the tooth-tissue at these points.

Let us examine the first of these claims. Is it true that angles are difficult to fill? A verdict based on years of practical experience by any operator who is observant will go to prove that so far as facility in building up a filling is concerned, the cavity which is formed of flat walls joined by angles is infinitely easier than a cavity formed of concave walls joined by curves. The flat-walled cavity with angles presents such a surface for the filling material to rest against that there is a sense of security in building the filling that never obtains where the condensation is against curved walls. As has already been intimated, there is little or no danger of the filling rocking under the impact of the plugger, as is so often seen when the walls are curved. The operator is not obliged to constantly watch his filling and retain it against tipping while condensing it, and the work may, therefore, be carried along more rapidly and with less tax on the operator. As to the possibility of gaining perfect adaptation of gold into the sharpest right-angle, there is no longer the slightest fraction of doubt. It has been demonstrated too many times to admit of argument. The only requisite is to have plugger points of the proper form, and then exert the force upon the gold in the right direction. The fact that gold will remain in position when driven into an angle more certainly than when driven into a groove or curve renders the operation much simpler where angles are formed, and a practical test of this matter will convince the sturdiest doubter.

As to the other question of the weakening effect upon the tooth by the formation of angles. Granted that in a general way a body will break more readily at an angle than at any other point, how many have seen teeth fracture along the lines where it is recommended that angles shall be formed in these cavities? The essayist has been a somewhat close observer of fractured walls of cavities, and he confesses that he has never yet found one located at these points. The place where the sharpest angle is suggested lies at the juncture of the gingival with the axial wall. Who has ever seen a tooth break here? Another point is where the axial wall joins the buccal and lingual walls, and instantly we may expect to hear a chorus go up to the effect that this is the very place where we repeatedly see fractures. On the contrary, I have never yet seen a single instance where a buccal or lingual wall has broken off along the line between it and the axial wall. I have noted very many cases where portions of these walls have burst away from a filling, usually leaving a more or less concentric fracture, the deepest portion of which does not, however, reach to the axial wall. The reasons for these fractures lie in an entirely different direction. They are usually caused by too deep grooving of the buccal or lingual wall, resulting in a thin edge to the enamel, which, not being supported by dentin, is readily fractured. Any spreading of the filling material under repeated impact in mastication may cause a bursting out of these thin edges, but the break seldom extends to the juncture of the broken wall with the axial wall, and it never passes any distance longitudinally along the line between these walls.

Another place where an angle is suggested is along the juncture of the base of the step with the surrounding walls of the step, and it would seem that occasionally a cusp might be lost as the result—particularly on an upper bicuspid. But a close observation of the condition of fractured bicuspids would appear to indicate that a break almost never occurs along this line. This entire subject of split and broken bicuspids is one of sufficient importance to demand a paper in itself, and it is worthy the most careful study of every practitioner, both with relation to the causes which bring about these fractures, and the means to be taken for their prevention.

But in the consideration of the present subject it must suffice to state that no danger of fracture need be feared from making an angle at the point indicated in a mesio-occlusal cavity in an upper molar. In short, it may be said that the principle of forming these cavities with flat walls joined by angles is a menace neither to the tooth nor the filling, but rather an element of safety to both, in the fact that a filling so placed is secure against movement under stress, and will not so readily leak or shift its position, and cause undue impingement upon a vulnerable wall of the cavity. The more securely we can lock our fillings into place, the more certain we may feel of permanent results.

As to the further details of cavity preparation—the form to be given the enamel margins, the instruments to be used, and the technique of the operation—the limits of the present paper will not permit us to consider. But before closing the subject, the statement should be made that no operator must feel discouraged if his attempt to perform these operations according to the principles laid down does not always meet with success. In every-day practice we encounter obstacles to the performance of ideal work, and we can not close our eyes to these obstacles. Some of them are too forceful to be ignored, and yet the danger with many operators is that a few obstacles are allowed to influence their entire line of practice, and that which should be the exception is turned into the rule. In other words, the first case that presents itself for our attention when we go back to our offices may prove to be one in which it is manifestly impossible for us to make an ideal preparation of the cavity through excessive sensitiveness of the tooth or nervousness of the patient. Too many operators under such a circumstance as this will simply throw up their hands and say that ideal cavity preparation is a myth, and not to be thought of in practical work. It is so convenient to stop short of perfection on the slightest provocation, and ease our conscience with the evidence of an exceptional case or two. This is all wrong, and it has a lowering effect on the profession. Our aim should be always to have the ideal before us and work faithfully and consistently toward that ideal. If we do this, our possibilities for attaining the ideal will widen perceptibly as we proceed, and in the end the cases in which we fail to reach the ideal will prove the exception rather than the rule. If all operators would approach their work in this spirit, it would place operative dentistry on a more serviceable basis, and result in a greatly improved class of work being accomplished for our patients. If this were done, we should no longer hear the intimation made by thoughtful men that the attempt to save decayed teeth by filling was in a large sense a failure. Some men in the profession have proved beyond the shadow of a doubt that the filling of teeth may be made successful, and what some men have done others can do, if they make the right kind of effort.

DISCUSSION OF DR. JOHNSON'S PAPER.

Dr. Callaghan: In reading Dr. Johnson's paper, the slow and very gradual growth of every branch of science is brought to my mind. As compared to other branches of science the art and science of filling teeth has been of rapid growth. From to me an unknown date lead, tin and gold have been packed by hand into tooth cavities to answer the purpose of stopping. In 1838 Dr. Merritt, of Pittsburg, used a hand mallet to condense gold after it had been inserted in place. In 1854 Dr. Robert Arthur called attention to the cohesive properties of gold foil. In 1860 Dr. W. H. Atkinson introduced the hand mallet for packing cohesive gold; this followed closely by Dr. C. R. Butler and others with improved instruments. In 1867 Dr. Bonwill, taking his cue from a telegraph sounder, constructed the electro-magnetic mallet.

It took the dentist hundreds of years, perhaps, to get up to the cohesive gold foil. It took twenty-two years to find what the hand mallet was good for. Then seven years later the electric mallet appears upon the scene, to be soon almost superseded by various forms of rapid mechanical mallets. By these rapid strides, with the aid of rubber dams, we are enabled to construct our beautiful and useful contour gold fillings. And now coming a few years later we are beginning to do the surgical part of the operation with some degree of skill and precision. Like other branches of our work, cavity preparation has developed from no preparation to the elaborate scheme set before us by Dr. Johnson. In the beginning of operative dentistry about the only theory of cavity formation was that "the bottom of the cavity should be larger than the orifice." Somewhere about

1838 ideas began to broaden, tooth cavities began to receive more elaborate preparation. Taft's Operative Dentistry, published in 1859, on page 153, we find the following direction for the preparation of anterior proximal cavities in bicuspids and molars: "If all the neighboring teeth stand in contact, separation cannot be accomplished by pressure, they must be in such cases separated wholly with the chisel and file, the interval should be large enough to enable the operator to manipulate with facility and see directly as possible into the cavity."

In 1877 while I for a short time was a pupil of Marshall Webb, removal of buccal and lingual walls to keep enamel margins free from contact with adjoining teeth, cutting of lingual wall below gum line and the beveling of margins were taught me.

As for the introduction of gold into a cavity we have not advanced one whit since 1877 and 1878. We, it seems to me, have reached the limits in cavity formation. With the publication of Dr. Johnson's book on filling the teeth, in 1900, or, in other words, cavity formation, comes twenty-three years later in the climax of gold building.

If you have followed Dr. Johnson carefully you will notice that he is decidedly conservative. He does not intimate that there is but one way or one set of details to be followed to properly prepare every cavity. He says, practically, let us see the cavity, study conditions and environments first, then keeping this ideal, if you please, in mind, proceed to the preparation of the cavity in the light of this, the condensed experience of the best operators of the world.

Full and free separation has been taught for many years, yet what percent of the mass of dentists do you suppose take the time to separate teeth properly. Not over 25 percent, you will say. The other 75 percent belong to the class of 1859, sixty years behind. How many of us trim away the bucco-lingual walls to the safety line? Not over 10 percent. The other 90 percent belong to the class of 1870, thirty years behind. Whether these estimates are correct or not, we do know that every day we see fillings the proximate walls of which are flat as boards, with decayed and broken down margins to such an extent that it makes one wonder that an intelligent public will tolerate such an imposition. We make a great effort to formulate and enact laws to regulate those about to enter our profession. It oftentimes strikes me that a law might be constructed and put into force that would do more for suffering humanity by operating internally rather than externally, as in the legal profession members of the bar who through professional misdemeanor lay themselves liable to disbarment.

How many of us make an honest effort to get a flat resisting gingival wall or border? I can not believe a sober dentist would deny the desirability of such a foundation; if there is, we will have to put him in the class of 1784 along with the French gentlemen who came to New York to practice dentistry. I must admit that at times I have been guilty of slighting the gingival wall. In the early days of my practice I depended entirely too much upon pits and grooves. Under the teachings of Black I moved up a step, and a mighty important step it was, too. It took me a long time to get rid of the grooving and pitting of this wall, but with the assistance of the proper use of cement and improved instruments a flat gingival wall has become a fixed habit. I believe with the essayist that the gingeval, axial and lateral walls should meet at sharp angles. The retaining grooves in the lateral walls have been and are to-day a source of annoyance to me for altogether too frequently I find myself making these grooves about twice the depth they should be. In the beveling and smoothing of enamel borders a sharp chisel is, of course, indispensable, but a round bur is oftentimes of great assistance. In this connection I beg to call your attention to the Ivory bur. For this purpose, to my eye, the small blades on this bur look as if they would leave the enamel surface rough, but an examination of the enamel borders under a glass will show them to be much smoother and more even than after the use of an ordinary spiral blade bur; they do not chip enamel to the same extent. In every respect I agree with Dr. Johnson on the subject as presented in his paper.

Dr. Harvey: I regret we have not seen Dr. Johnson's operation so we might discuss that in connection with the paper, as I had anticipated. He first gives us reasons for presenting this paper, which I hope is not in the nature of an apology, for it is one that interests us and is of great importance to us. In the beginning of preparation of cavity the first important thing spoken of is the necessary space between proximate teeth. The object, as I take it, in the preparation of these cavities, in fact all cavities, is the insertion of a filling of some material which would restore tooth to a typical form and the preservation of its usefulness. It may not always be necessary, however, to gain this space previous to preparation of cavity, but we should never proceed with filling until we have gained sufficient space. I should like to emphasize that proceeding. It is fair to presume that the large percentage, or quite a percentage of the cavities occurring in this locality, when they become mesio occlusal, are quite extensive, and in speaking of these cavities I will assume it is a large cavity. If it is such, then I will agree entirely with Dr. Johnson's description and method of preparing outline that is to be carried beyond the contact point with protruding teeth and also cutting away any frail gingival wall and paring it away below gum. But if these cavities are not so large, and especially in the younger patients, I should hesitate to cut that cavity very much beyond the point where I get good, secure, solid walls against which to build the gold.

Dr. Johnson is not radical in his ideas on this point, and has given us a good deal of margin for the use of our judgment. He next refers more particularly to the form of the interior of the cavity for the purpose of retaining filling. I presume from his description of the operation he would start his filling either with soft gold, or at least an annealed gold, and finish with cohesive. If that is his method, I should agree entirely with his formation, believing these point angles are very much better than grooves or pits. Point angles need not be extended to undermine the enamel and need not go higher than the step formation for the retention. However, if the cavities are very much extended bucco-lingually, or for any reason they are shallow from margin to axial wall, either from filling in with cement or naturally, we have a broad extent of surface to cover, and if we wish to start with cohesive gold I would advise using a pit—I do not mean a large hole, but a small pit to keep the gold from rocking until we can bridge across. These pits should be small, made with drill of small point and should not be deeper than the diameter of pit. There are cases where these are an advantage to enable us to use cohesive foil and to make better start.

The formation of the step for the anchorage is made, of course, on mechanical principles, where we get the greatest strength for holding and anchoring it at the top, for this, of course, there should be undercut or point angles. It is not necessary to extend entirely around the margins, but simply sufficient to retain gold. The doctor also says, make the bucco-lingual walls perpendicular as far as possible. This is very well, as we can condense gold much more certainly to obtain good, strong edge to filling than in any other form. He has left out the beveling of enamel walls. I do not think he means that the walls should be perpendicular clear to the enamel edge. They should be beveled sufficient to condense gold to the enamel margin. The great value in the paper presented by Dr. Johnson to me, and all of us, in summing up, is that it gives us an ideal to work forward to; as brought out in the close of his paper. We can not arrive at good results unless we have an ideal, and the nearer we approach to it the better, but our judgment should always be used as to how far we progress toward that ideal.

Dr. H. T. Smith: Dr. Johnson spoke about the *more* permanent fillings. He is cautious about speaking about permanent fillings. He says extension for prevention is not a permanent operation, hence uses the term "more permanent." I think any experienced practitioner who is a close observer knows the doctrine promulgated by Dr. Black and carried on by Dr. Johnson, is the proper procedure in the preparation of a cavity. No man who studies dental caries at the chair can admit of any other method. If you study the beginning of decay on the proximate surfaces, it is well known that it is better settled than the etiology of decay in any other locality. That is a broad statement, but I think it is true. Miller demonstrated it. Miller says no acid, no decay; and this acid is due to a simple local affection. In the preparation of that cavity unless you improve the environment, unless you prevent the possibility of the development of an acid, you have not done much good, and since the teeth are nearly always vulnerable at certain points and usually the same points, if you do not improve this condition you do not do much good. Dr. Wright insists that even though we do this it does not necessarily make it a permanent filling, nor does it. It is the idiosyncrasy of the individual or the secretion of saliva which causes the decay. Dr. Stephan made a statement which is important, I think, in reference to contour of the teeth, insisting upon proper contour. He said the nearer you brought the tooth to its original shape the better. There are, however, exceptions to this. It may depend upon conditions of surrounding teeth and their contour. Take, for example, if we have a cuboidal molar tooth, the proper shape is more nearly square. We should correct that form; they decay more easily of that form. Horses' teeth are of this shape with large and flat surfaces, but they do not decay, but human teeth of such shape decay very quickly. Now, in such cases we do not want to restore the original form, but improve upon it; that is one reason we want extension for prevention. Take again a well rounded bicuspid and, if restored to its original shape, it will be the best filling possible, but if you leave flattened surfaces and bring adjoining teeth into close contact with it, the condition is not improved. I do not like to say that operative dentistry is a failure, but it is largely so because we do not prepare our cavities along the proper line, assuming, of course, that the filling is made as good as possible. We want to reduce the failures to a minimum and if you follow out the reasons for so doing, as presented here to-day, you are adopting the right procedure.

Dr. Ruggles: A man without an ideal and a purpose makes very little progress. We must have an ideal in order to get results. We all know we can not form each cavity to the ideal mentioned in this paper, but we can nearly do so; we can at least restore proximate contact, if it is advisable. There is one position where we should try to have that as near normal as is obtainable, and that is near the occlusal surface. In order to do that we must be familiar with dental anatomy to form the lines upon the proper rules and make filling accordingly. Dr. Johnson is a close student of Dr. Black, therefore his principles and also his nomenclature are faultless, and the latter is one of the things in which dentists, as a rule, are deficient, and they can not take up a subject for discussion because they do not know the nomenclature. If we could take this paper home and read it, and make ourselves familiar with all the terms, disto buccal, axial, mesio occlusal, etc., we would all be better off.

Dr. Whitslar: About the terms permanent and temporary, I think these are simply relative terms; it depends entirely upon the case at hand, and this requires extreme judgment upon our part; in fact, in the main we may say, judgment is the prime factor in the treatment of diseased teeth. What do we fill teeth for? In the main for the sake of restoring function of the teeth, and secondly for cosmetic effects, and we also go further in trying to overcome bad functions or to better their condition. We may find it necessary, to get proper use of the teeth, to extend the contour of teeth beyond the ideal limits in order to make them occlude properly. There are many things upon which the success of an operation depends; constitution of patient, and, as Dr. Cassidy yesterday brought out in his paper, the chemistry of the nerves; it also seems that the secretion of the mouth has much to do with decay of the teeth, and these secretions may be affected by disease of the nerves, or may be changed after coming into the mouth. So for these various reasons the terms permanent and temporary can only be relative. I am very much in favor and want to indorse the ideas that Dr. Johnson has given us in regard to extension for prevention; yet these terms also have their limitation, depending upon the individual entirely; we must also in these cases use judgment.

Dr. Taft: As to method suggested, I have no criticism to make any further, perhaps, than that almost everybody can find some fault with everything. It is always better, however, to give every man the benefit of the doubt. One or two suggestions. In regard to the formation of cavities. The angles spoken of to be formed at cervical portions of cavity, buccal and lingual walls, I have used them for a good many years, they should not be acute angles, a right angle is quite sufficient in most cases for introduction and retention of filling. But, as stated in paper today, it may be poorly filled. Many years ago the form of cavity just at this point was accomplished by drilling a little pit or cavity and serious mischief was often done by making the pit too large, undermining the enamel and weakening the border. In forming the buccal and lingual walls of the mesial and distal cavities in incisors, and especially where the large surfaces approximate, he spoke of cutting in towards the central or axial wall. This is practical in many cases, but the novice will many times cut in through the dentin to the enamel, in no instance should the dentin be cut through to the enamel. The danger lies in the filling of the cavity. A wrong adjustment of the plugger will break the enamel wall out. This matter of forming cavities is one of the most delicate operations, it is comparatively easy to introduce a filling after the cavity is well formed. Judgment should always be used that the cavity to be so formed as to allow easy manipulation of gold. This matter of having the cavity always lined with dentin, or rather, refusing to cut away dentin when the enamel would be exposed, is a vital one. Reference was made to the self-cleansing fillings. This is a very important matter.

Some one stated we should always restore a tooth to its original form. This is possible if the adjacent teeth are present and tooth is of regular shape, and if irregular teeth have been extracted, leaving the remaining teeth bridging over into the space, larger contour may be necessary. Every case must be examined carefully and filled according to best judgment. A good filling may then be accomplished.

Dr. Johnson: The day of torturing our patients with needless separation or wrong methods of separation is passed. We can obtain all the space that is necessary for these operations without subjecting patient to any pain whatever. I use gutta-percha and pack it in between the teeth; this is ideal for that separation. I want to raise a point of caution against the use of India rubber. The average patient will always come to you with a dread of having teeth separated. India rubber will not remain stationary upon the surface of teeth: it will creep root-wise and get into the inter-proximal space. It is painful and sets up soreness of teeth. If I were the patient I wouldn't have it in my mouth, I never find it necessary, but I don't want to become prejudiced against any line of practice. If I should place rubber between teeth I would build a wall of gutta-percha from gingival third of cavity against the tooth next in line, so it would form a bridge and prevent crowding on the gum tissue. In other cases which come to us we can get the required separation by the use of separator at the time. It is an easy method if used used rightly. We must always recognize the force the separator is making and to regulate it with intelligence. In regard to stopping short of extreme extension, why I should go home ashamed of myself if I was supposed to extend all cavities to the extent which I did here this afternoon. If there is anything I pride myself on it is good judgment. In a young patient, sixteen years of age, it would be criminal malpractice to extend the cavity for anything like a permanent operation, and the same with a nervous boy or girl. There is something infinitely more important than to make a permanent operation in these cases; we should so handle that child as to gain his confidence and not mistreat him, and thus keep him from a dentist's office ever afterwards. We should study all the surroundings of the operation, character of person, etc.; but many times we stop short of this. But whenever we do this it must be for the definite purpose of the case; and, with modern methods of manipulation, and intelligent digital manipulation, we can accomplish ideal results in a great many cases. If I see a patient, eighty years of age, who has a filling in a lateral incisor which has been there for more than sixty years, I am willing to call that a permanent filling. A dear old mother of Israel once took my chair for a minor operation, and she said: "See that filling; it was put in there more than sixty years ago." It was a splendid filling and will remain there until she dies. I wish I knew the man who performed that operation. I would travel across the continent to congratulate him. If it was possible for men sixty years ago to have done this work, it is possible for us to-day.

If I do not feel within my inmost soul that some of my fillings will last as long as I do, I should feel as if I were a failure.

In regard to the extension of contour of tooth to original form, have been pleased with what I have heard. I do not state any particular case with the idea of bringing about a certain ideal form, but always to restore to the best original form. In many cases I improve upon the form which has been given. In a case which I had where a lady had lost the first permanent molar on lower jaw (and by the way, I wish the profession would rise to the necessity of saving the first permanent molar; it is the keystone of the arch), the second molar had tipped forward and the second bicuspid leaned backward, the distal surface of bicuspid being badly decayed. The dentist who had charge of the case would not make that filling because it was too difficult. In studying case I saw if the trouble were to be rectified it would be necessary to bridge across that space and have filling come into contact with molar. The tooth was badly broken down disto-lingually; I explained to patient it would be a long, tiresome operation. She said she was glad and willing to undergo the operation to have the tooth repaired, as it was the worst tooth in her mouth, and it was almost impossible for her to eat comfortably. Filling was made, contoured so as to extend to contact with molar, and finished. The gum tissue in a short time filled up perfectly between these teeth as in any normal interproximal space.

Dr. Taft in his remarks opened up one of the most important subjects to be considered in operative dentistry, in regard to the difference in the structure in teeth. Every practicing dentist recognizes the difference between the structure of one tooth and another; some cut easily with chisel and bur, while with others it is almost impossible to break down. Now this is not so much a difference in the structure of the teeth, so far as the constituents of the teeth are concerned, but a difference in the relation of the enamel rods one to another. If these two classes of teeth were analyzed chemically, you will find as large a percentage of lime salts in the one as in the other. This difference is simply due to the arrangement of the enamel prisms and not the chemical difference in structure. In some cases the prisms run more regularly and radiate straight out upon the dentin, one beside the other, and the cement substance which holds these prisms together is easily broken down. In other cases these prisms are wavy, irregular, and held firmly together, and it would be necessary to break across a prism in order to fracture the structure. But the truth is, that teeth which are so resistant to our instruments may be just as readily attacked and broken down by the cause of caries as would be a poorly constructed tooth. The point I wish to make is this, it is more a question of environment, the conditions surrounding the teeth, than tooth structure itself, which makes the difference in susceptibility to decay. A tooth may be perfectly developed and yet be quite susceptible to caries; and again, the tooth may be friable and break down very easily, and yet the mouth may be almost free from decay. I have seen this time and again. Have had cases in same family where equal attention was paid to teeth, and they were equally developed, and yet in one case the teeth break down and decay very readily, and the other almost free from caries. There are also periods in which we find decay of the teeth going on more rapidly in the same mouth. These cases are very discouraging at times, but let us go to work and fight back at the case, make temporary operations if necessary, and in nine times out of ten if the work is carried on diligently and with intelligence the cruel destroyer will be conquered and decay will cease. Dr. Taft mentioned a case where a plug of wood stopped decay of tooth. Why, I have seen cases where decay was stopped without anything being placed in the cavity. I wish we could all study immunity and susceptibility; it is our greatest hope in holding back decay. I hope you will pardon me if I refer to my book; it is something I never do, but as it has been referred to I would like to request you to read the chapter on dental caries, page 31, in which the case I am going to state was published. This was a case of a young girl whose teeth were very badly decayed and decay was going on very rapidly and extensively. Her incisor teeth were good, but the posterior teeth were nearly all gone. Very few dentists would have had any hopes of saving them. I went to work, plastered them all up with amalgam; it was the most abominable sight possible. After struggling for some time, filling and refilling with such filling, a year finally elapsed, and she did not appear again; I though, well, Clara has given it up. One day she came into the office smiling; she had developed into a most beautiful girl, and she said she had had no further trouble with her teeth, but she thought it best to have them examined. On examining the teeth I found they had not decayed to any great extent, and to-day she is a young lady of about twenty-one or twenty-two years of age, and she has all her teeth, and will have as long as I live.

One word in conclusion: When we are filling teeth we ought to be doing something more than simply standing to one side of patient and plugging that little hole with some filling material. We should study the environment of the teeth, the surrounding gums, interproximal space and functional activity of the teeth on that side of the mouth. Get out of the habit of simply filling these little holes and thinking that it is a minor operation.

Mr. President, I can not close without offering a sincere word of thanks to this Society and for the kind reception given me here to-day. I have known many of the members for years. I deem it a special privilege to be here at this time. Probably I would have done better if I had been attacked a little more severely, for I bite badly. It is just such occasions as these that cause men to go on and work a little bit harder than before. There are twenty-four hours in every day, sixty minutes in every hour and sixty seconds in every minute, and you can accomplish something in every second, and it is just such occasions as this that make one ambitious to accomplish something every second.

---

The British House of Lords has passed an act preventing the organizing of companies for carrying on the practice of dentistry which unregistered persons are now able to do by forming a corporation of at least seven members, none of whom need be educated or skilled dentists.

